# Mitochondrial *COX3* and tRNA Gene Variants Associated with Risk and Prognosis of Idiopathic Pulmonary Fibrosis

**DOI:** 10.3390/ijms26031378

**Published:** 2025-02-06

**Authors:** Li-Na Lee, I-Shiow Jan, Wen-Ru Chou, Wei-Lun Liu, Yen-Liang Kuo, Chih-Yueh Chang, Hsiu-Ching Chang, Jia-Luen Liu, Chia-Lin Hsu, Chia-Nan Lin, Ke-Yun Chao, Chi-Wei Tseng, I-Hsien Lee, Jann-Tay Wang, Jann-Yuan Wang

**Affiliations:** 1 Department of Laboratory Medicine, Fu Jen Catholic University Hospital, Fu Jen Catholic University, New Taipei City 24352, Taiwan; linalee@ntu.edu.tw; 2 Department of Internal Medicine, Fu Jen Catholic University Hospital, Fu Jen Catholic University, New Taipei City 24352, Taiwan; chouwenru@gmail.com (W.-R.C.); pforcekuo@gmail.com (Y.-L.K.); cychang627@gmail.com (C.-Y.C.); 3 School of Medicine, College of Medicine, Fu Jen Catholic University, New Taipei City 242062, Taiwan; medrpeterliu@gmail.com; 4 Department of Laboratory Medicine, National Taiwan University College of Medicine and Hospital, Taipei 10051, Taiwan; jennifer_jan5363@yahoo.com.tw (I.-S.J.); curt3333cc@gmail.com (H.-C.C.); 5 Department of Critical Care Medicine, Fu Jen Catholic University Hospital, Fu Jen Catholic University, New Taipei City 24352, Taiwan; qqcu111@gmail.com; 6 One-Star Technology, New Taipei City 11051, Taiwan; ringu3333@gmail.com; 7 Department of Internal Medicine, National Taiwan University College of Medicine and Hospital, Taipei 10051, Taiwan; clhsu7@ntu.edu.tw (C.-L.H.); wangjt1124@ntu.edu.tw (J.-T.W.); 8 Department of Medical Imaging, Fu Jen Catholic University Hospital, New Taipei City 24352, Taiwan; b9302022@gmail.com; 9 Department of Respiratory Therapy, College of Medicine, Fu Jen Catholic University, New Taipei City 24352, Taiwan; 148540@mail.fju.edu.tw; 10 Cardiovascular and Pulmonary Rehabilitation Center, Fu Jen Catholic University Hospital, New Taipei City 24352, Taiwan; 11 Department of Respiratory Therapy, Fu Jen Catholic University Hospital, New Taipei City 24352, Taiwan; kiwitseng724@gmail.com

**Keywords:** cytochrome c oxidase subunit 3 (COX3), idiopathic pulmonary fibrosis (IPF), mitochondrial DNA (mtDNA), nonsynonymous variant, tRNA variant

## Abstract

Idiopathic pulmonary fibrosis (IPF) has been associated with mitochondrial dysfunction. We investigated whether mitochondrial DNA variants in peripheral blood leukocytes (PBLs), which affect proteins of the respiratory chain and mitochondrial function, could be associated with an increased risk and poor prognosis of IPF. From 2020 to 2022, we recruited 36 patients (age: 75.3 ± 8.5; female: 19%) with IPF, and 80 control subjects (age: 72.3 ± 9.0; female: 27%). The mitochondrial genome of peripheral blood leukocytes was determined using next-generation sequencing. During a 45-month follow-up, 10 (28%) patients with IPF remained stable and the other 26 (72%) progressed, with 12 (33%) mortalities. IPF patients had more non-synonymous (NS) variants (substitution/deletion/insertion) in mitochondrial *COX3* gene (coding for subunit 3 of complex IV of the respiratory chain), and more mitochondrial tRNA variants located in the anticodon (AC) stem, AC loop, variable loop, T-arm, and T-loop of the tRNA clover-leaf structure in PBLs than the control group. The succumbed IPF patients were older, had lower initial diffusion capacity, and higher initial fibrosis score on high-resolution computerized tomography (HRCT) than the alive group. NS variants in mitochondrial *COX3* gene and tRNA variants in PBLs were associated with shorter survival. Our study shows that (1) leukocyte mitochondrial *COX3* NS variants are associated with risk and prognosis of IPF; (2) leukocyte mitochondrial tRNA variants located in the AC stem, AC loop, variable loop, T-arm, and T-loop of the tRNA clover-leaf structure are associated with risk, and the presence of tRNA variants is associated with poor prognosis of IPF.

## 1. Introduction

Interstitial lung disease (ILD) is a general term that includes diseases with diffuse fibrotic foci in pulmonary interstitial tissue, with or without inflammatory cell infiltration [1,2]. Among them, the major disease entity is idiopathic pulmonary fibrosis (IPF). IPF occurs mainly in elderly smoking men, with most of patients being 60 to 75 years old at diagnosis [3,4]. On high-resolution computerized tomography (HRCT), IPF is characterized by sub-pleural reticular opacities, honeycomb appearance, and traction bronchiectasis, mainly in lower lobes and sub-pleural regions [1,2,3,4]. The median survival of IPF patients is 2.5 to 3.5 years [3,4]. The development of IPF is related to chronic inflammation and repeated injuries to the alveolar epithelium. At sites of epithelial injury, platelets enter and produce growth factors such as transforming growth factor beta 1 (TGF-β1), platelet-derived growth factor (PDGF), epidermal growth factor (EGF), fibroblast growth factor (FGF) and cytokines interleukin (IL)-4 (IL-4) and IL-13, attracting inflammatory cells into the injury site and causing mesenchymal activation. The latter causes resident fibroblast expansion and differentiation into myofibroblasts [5,6]. Under the normal healing condition, activated myofibroblasts induce granulation tissue and subsequently undergo apoptosis. When the healing process is dysregulated, activated myofibroblasts cause deposition of excessive extra-cellular matrix and fibrosis, such as in IPF [5,6,7,8]. What mechanisms deviate the healing process to fibrosis is elusive.

Mitochondrial DNA (mtDNA) damage has been shown to induce alveolar epithelial cell apoptosis with subsequent fibrosis by producing excessive reactive oxygen species (ROS) [9,10,11]. Mitochondrial dysfunction, including decreased mitochondrial respiration and increased ROS production, was associated with initiation and progression of pulmonary fibrosis in mice [12]. In IPF patients, damaged mitochondria with dysregulated autophagy and decreased mitochondrial electron transfer chain (ETC) activity have been found in alveolar epithelial cells [13] and fibroblasts [14], and plasma mtDNA concentration was significantly increased and associated with IPF progression [15].

Thus, damage and dysfunction of mitochondria can be associated with development and progression of pulmonary fibrosis. Yet mtDNA variants, which may result in mitochondrial dysfunction, were rarely investigated in patients with IPF. Mitochondria have their own DNA, separated from the nuclear genome. Human mtDNA is a circular double-stranded DNA (one heavy and one light strand) of 16,569 base pairs [16]. Although the majority of mitochondrial proteins are encoded by the nuclear genome, mtDNA does code 13 important mitochondrial proteins (including part of complex I, and all of complex III, IV, and V of the ETC), 2 ribosomal RNAs (rRNAs), and all 22 mitochondrial tRNAs. The 13 mtDNA coded proteins are transcribed and translated by mitochondrial rRNAs and tRNAs [17]. Thus, variants in the mitochondrial genome may affect synthesis of ETC proteins and mitochondrial function.

We hypothesized that mtDNA mutation, deletion, or insertion that could potentially affect mitochondrial function, may increase the risk of IPF. Instead of investigating mtDNA variants in pulmonary cells, we performed the analysis on peripheral blood leukocytes (PBLs). No study has been reported on mtDNA variants in PBLs of IPF patients. Yet in mice, environmental molecules that enter and affect alveolar epithelial cells can disrupt the basement membrane, leading to large gaps with denuded basement membrane, as shown by electron microscopy. These molecules can accumulate at the gaps, penetrate the very thin endothelium of the capillary, and enter blood circulation [18]. Thus, PBLs and alveolar epithelial cells can be exposed to similar environmental molecules that may cause mtDNA damage and increase the risk of IPF. In the current study, we used next-generation sequencing (NGS) to determine the entire mtDNA genome of PBLs, and investigated the effects of mtDNA variants in PBLs on the risk and prognosis of patients with IPF.

## 2. Results

A total of 36 patients (women: 7 [19%]; age: 75.5 ± 8.4 years) with IPF participated in the study (Table 1). The control group consisted of 80 subjects (women: 22 [27.5%]; age: 72.3 ± 9.0 years). There were no differences between the two groups on age, sex, race distribution, history of smoking, history of drinking, and co-existing diseases.

All the 36 IPF patients presented with varying degree of cough, shortness of breath, dyspnea on exertion, and basal crackles. The mean initial fibrosis score was 8.6 ± 2.3 (range 6–14). The mean initial forced vital capacity (FVC) (as % predicted) was 75.4 ± 26.2, and mean initial diffusion capacity for carbon monoxide (DLCO) (as % predicted) 69.4 ± 24.0.

Among 36 IPF patients, 11 (31%) received two anti-fibrotic drugs sequentially (nintedanib followed by pirfenidone [N = 6], or vice versa [N = 5]), due to disease progression or adverse drug effects. Fifteen (42%) patients received nintedanib only, and 8 (22%) pirfenidone only. Two (5%) patients did not receive any anti-fibrotic drugs (one each due to mild symptoms or irregular follow-up). During the follow-up period ranging from 12 to 45 months, 21 (58%) patients progressed (change of predicted FVC, −25.8 ± 31.3%), and 15 (42%) remained stable (in 13 to 80 months after diagnosis; change of predicted FVC, 0.87 ± 5.9%, *p* = 0.003). At the end of the follow-up period, 12 (33%) IPF patients and six (8%) in the control group died (*p* = 0.001). Median survival of the 12 succumbed IPF patients from the diagnosis was 19.5 months (range: 4 to 72 months). Causes of death in the IPF group were IPF progression in 11 and diabetic foot with sepsis in one, and in the control group pneumonia (N = 3), stroke (N = 2) and pemphigus with septic shock (N = 1).

### 2.1. Mitochondrial DNA Variants

**Figure 1:**
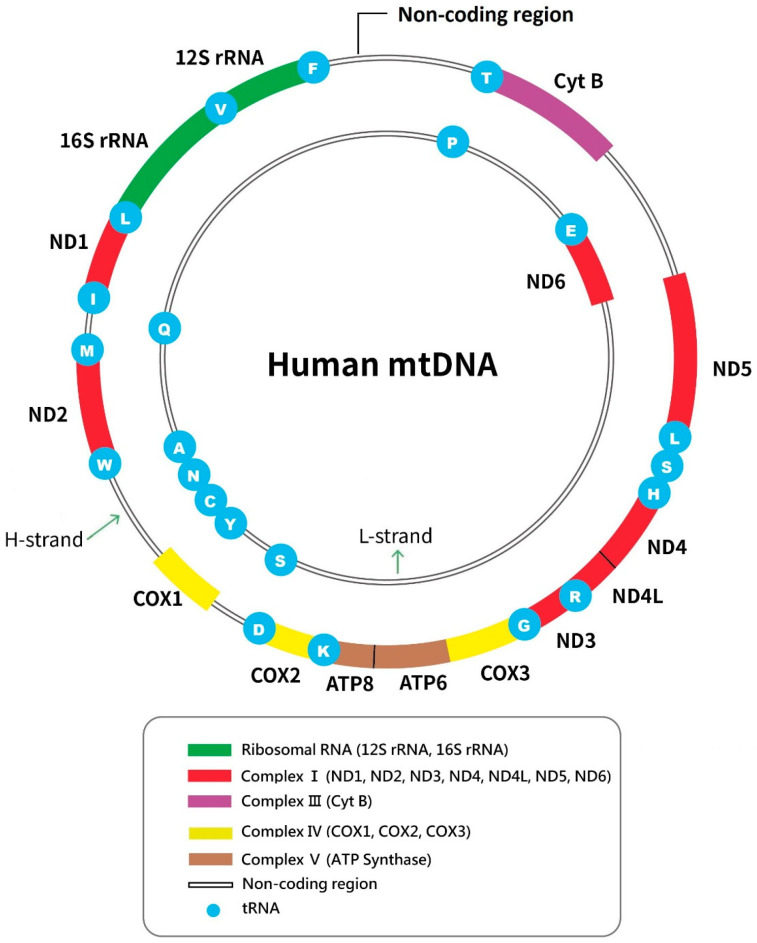
The map of human mitochondrial DNA, a double-stranded circular DNA with H- and L-strands. It has 37 genes coding for 13 proteins of the respiratory chain (complex I, III, IV, and V), 2 rRNAs, and 22 tRNAs.

We found 1457 and 3245 variants in 775 nucleotide positions in the IPF and control group, respectively (average 39.9 ± 9.0 and 40.6 ± 7.3 per case, respectively, *p* = 0.659; Table S1, Supplementary Materials). The nucleotide variation rate was 4.68% (775/16,569). The average number of total non-synonymous (NS) variants (substitution/deletion/insertion) of the entire mitochondrial genome per case was also similar in the IPF and control group (8.9 ± 2.4 vs. 8.8 ± 2.5, *p* = 0.796). However, 8 (22%) IPF patients had in total 9 NS variants in the *COX3* gene (coding for cytochrome c oxidase subunit 3 of the respiratory chain complex IV) (Figure 1), compared with 8 NS variants in the *COX3* gene in 80 (10%) control subjects (22% vs. 10%, *p* = 0.148). Yet the average number of *COX3* NS variants per patient in the IPF group was significantly higher than in the control group (0.3 ± 0.5 vs. 0.1 ± 0.3, *p* = 0.048). Of these variants, in the IPF group five were insertions, two deletions, and two NS substitutions, compared with 2, 1, and 5, respectively, in the control group (Table 2).

**Figure 2:**
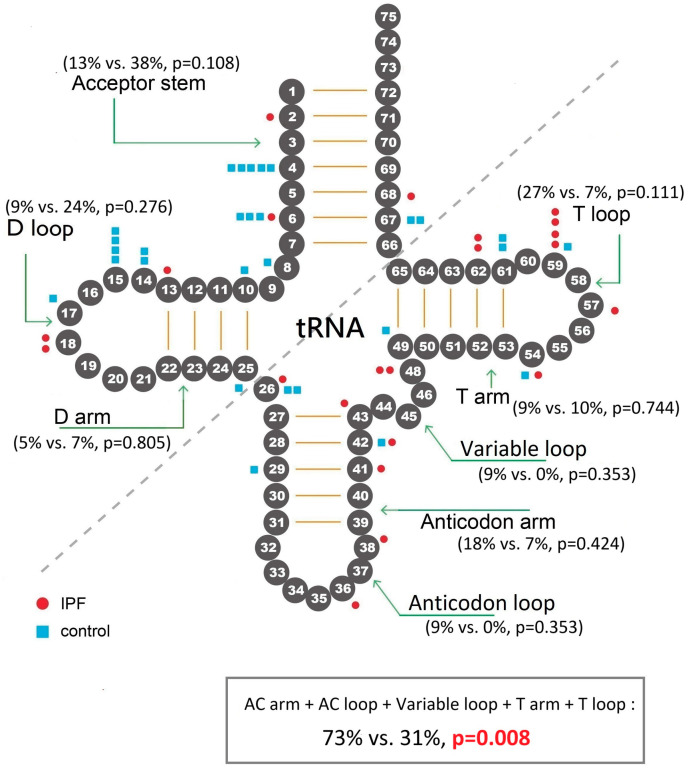
Distribution of mitochondrial tRNA variants in regions of the secondary clover-leaf tRNA structure. Patients with IPF had more tRNA variants located in the right half of the clover-leaf structure (including anticodon loop, anticodon arm, variable loop, T-arm, and T loop) than the control subjects, with the majority of tRNA variants located in these regions (73% vs. 31% in the control group, *p* = 0.008). Numbering of nucleotides follows the standard system [19].

As for the 22 mitochondrial *tRNA* genes (Figure 1), we found that there were 22 *tRNA* variants in 15 (42%) of the IPF and 29 *tRNA* variants in 25 (31%) of the control group (42% vs. 31%, *p* = 0.346). Of the 15 IPF patients with tRNA variants, four also had *COX3* gene NS variants, compared with four in 25 control subjects with *tRNA* variants (4/15 or 27% vs. 4/25 or 16%, *p* = 0.502). The average number of mitochondrial *tRNA* variants per patient in the IPF group was higher than the control group, although not statistically significant (0.6 ± 0.9 vs. 0.4 ± 0.6, *p* = 0.077; Table S1, Supplementary Materials). Table 3 shows details of *tRNA* variants, which occurred in all regions of the mitochondrial tRNA clover-leaf secondary structure (Figure 2). The distribution of *tRNA* variants in these regions was different between the IPF and control group (Table 3; Figure 2). Among the 22 *tRNA* variants in the IPF group, there were 16 (73%) located in the anticodon (AC) arm, AC loop, variable loop, T-arm and T loop (the right half of the clover-leaf secondary structure), significantly more than the control group (9/29 or 31%, *p* = 0.008). The average number of *tRNA* variants in these regions per person was also higher in the IPF than in control group (0.4 ± 0.8 vs. 0.1 ± 0.3, *p* = 0.004).

### 2.2. Survival Analysis and Risk Factors for Mortality

We compared clinical characteristics and mtDNA variants between the succumbed and alive group of IPF patients (Table 4). The succumbed patients were older (80.7 ± 6.7 vs. 72.9 ± 8.1 years, *p* = 0.007), with comorbidities similar to the alive group. The initial DLCO, but not FVC, of the succumbed patients was lower, and the initial HRCT fibrosis score of the succumbed group was higher than that of the alive group. The average number of *COX3* gene NS variants per patient of the succumbed group was significantly higher than that of the alive group (0.5 ± 0.7 vs. 0.1 ± 0.3, *p* = 0.032), and so was the average number of *tRNA* variants of the succumbed vs. the alive group (1.3 ± 1.2 vs. 0.3 ± 0.5, *p* = 0.002).

**Figure 3:**
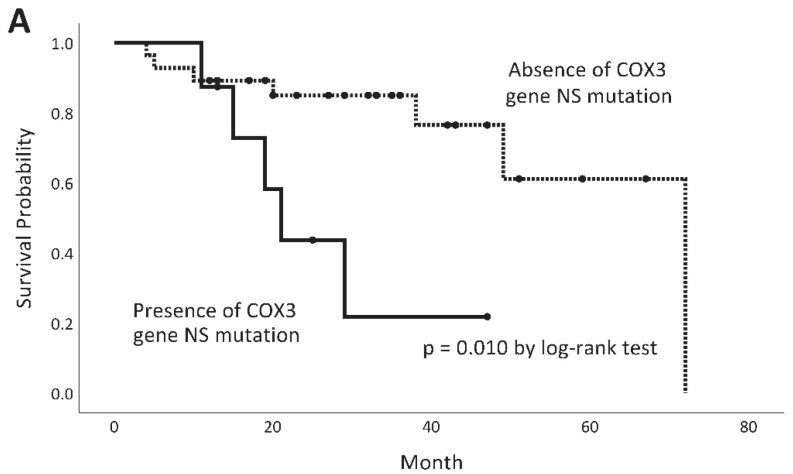

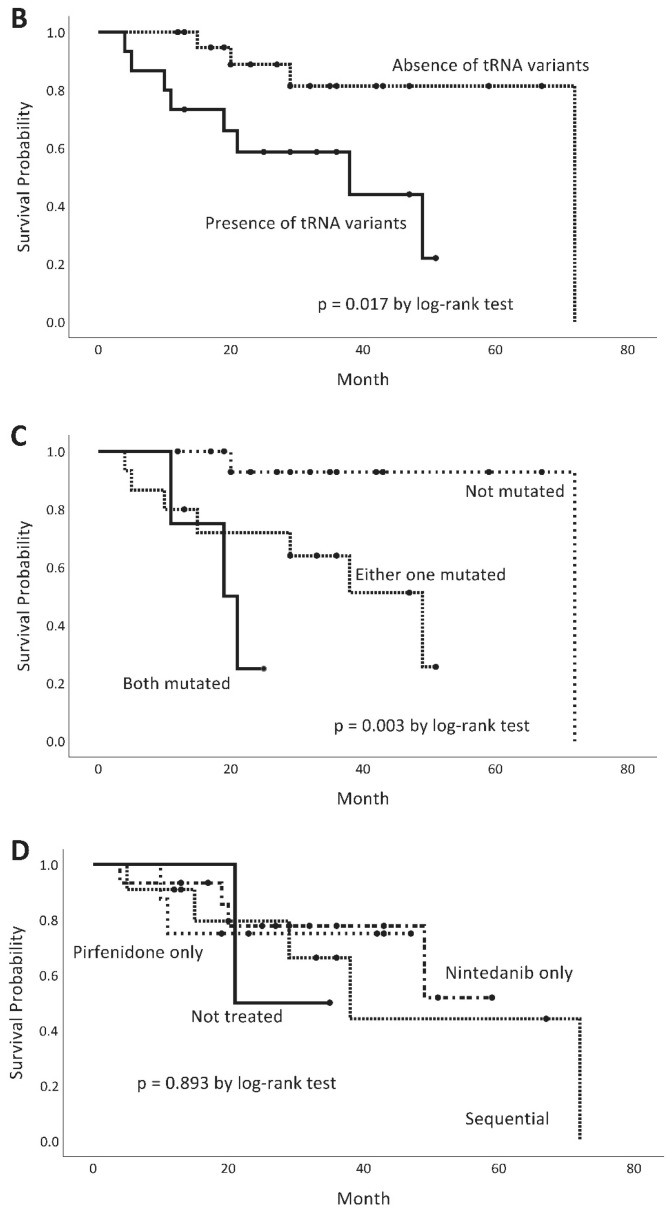
Survival curves of patients with IPF, stratified by mitochondrial *COX3* gene NS variants (substitution/deletion/insertion) (**A**), mitochondrial tRNA variants (**B**), the presence of neither, either, or both *COX3* gene NS variants and mitochondrial tRNA variants (**C**), and anti-fibrotic treatment (**D**). For the 3 curves in (**C**): *p* = 0.127 when comparing between “Both mutated” and “Either one mutated”; *p* = 0.011 when comparing between “Either one mutated” and “Neither mutated”; *p* = 0.001 when comparing between “Both mutated” and “Neither mutated”. The four curves in (**D**) are: IPF patients treated with pirfenidone only, nintedanib only, pirfenidone and nintedanib sequentially (or vice versa), or not treated.

Log-rank test shows that IPF patients with *COX3* gene NS variants had poorer survival probability than those without (*p* = 0.010), and so did those with *tRNA* variants vs. those without (*p* = 0.017) (Figure 3A,B). IPF patients who had neither *COX3* gene NS variants nor tRNA variants had the best survival probability (Figure 3C). The four IPF patients with both *COX3* gene NS variants and *tRNA* variants had survival probability not different from those with either one alone (Figure 3C). IPF patients who received nintedanib only (N = 15), pirfenidone only (N = 8), the two drugs sequentially (either nintedanib to pirfenidone or vice versa, N = 11), or no anti-fibrotic drug (N = 2), had similar survival probabilities (Figure 3D).

We analyzed risk factors associated with mortality using Cox proportional hazards regression. Variables considered in the statistical model included age, sex, smoking status, initial FVC, HRCT fibrotic score, *COX3* NS variant, and *tRNA* mutation. We discovered six risk factors associated with survival: age, current smoker, initial FVC < 60% predicted, initial HRCT fibrosis score > 8, *COX* NS variants, and *tRNA* mutation (Table 5).

## 3. Discussion

The prospective study had two main findings. First, compared with the age- and sex-matched control group, patients with IPF had (A) similar co-morbidities, higher mortality, and more mitochondrial *COX3* gene NS variants (substitution/deletion/insertion), which lead to amino acid substitution, truncation, or a completely different protein of the COX3 subunit of cytochrome c oxidase; (B) more mitochondrial *tRNA* variants that were located in the AC arm, AC loop, variable loop, T-arm, and T loop than the control group. Second, compared with the alive group, the succumbed IPF patients had more *COX3* gene NS variants and more *tRNA* variants. IPF patients with either *COX3* gene NS variants or *tRNA* variants had poorer survival probability than those without.

Mechanisms leading to IPF likely involve many pathways. Previous studies have discovered that variants in genes coding for mucin, a telomerase protein, or surfactant were associated with increased risks of fibrotic interstitial lung diseases [20]. However, these are variants in nuclear genome. The association between IPF and mtDNA variants have been rarely investigated. IPF is more common in the elderly [21], and aging has been found to affect mitochondrial morphology [22,23] and function [14,24]. Moreover, TGF-β1, the wound healing promoting cytokine that induces differentiation of fibroblasts into myofibroblasts with extracellular matrix overproduction [25,26], has been reported to up-regulate mitochondrial mass in fibroblasts [27]. Thus, mitochondria are involved in both aging and TGF-β1-related abnormal would healing, the two factors contributing to IPF.

Our study revealed that compared with the control group, IPF patients had more NS variants in the mitochondrial *COX3* gene, and those with these variants had poorer survival. The mitochondrial *COX3* gene codes for the 3rd subunit of cytochrome c oxidase (COX), i.e., complex IV of ETC [28]. ETC consists of four enzyme complexes: I, II, III and IV. The energy-generating process of mitochondria is initiated at complex I or II where nicotinamide adenine dinucleotide (NADH) or flavin adenine dinucleotide (FADH2) are oxidized, and electrons are released. The electrons are transferred to complex III and IV, and in complex IV they combine with oxygen to form water. In the electron transfer process, proton (H^+^) electrochemical gradient is built and utilized by complex V to generate ATP [28]. Thus, COX is the terminal and rate-limiting enzyme in the ETC.

We found that of the nine *COX3* gene NS variants that occurred in eight IPF patients, five were insertions and two deletions, leading to frame shift and abnormal or truncated COX3 protein. But because these *COX3* gene variants were heteroplasmic, patients harboring the variants probably still had normal COX3 protein [29]. Yet the quantity of normal COX3 protein, the function of complex IV, and the efficacy of ETC would probably be decreased. In the literature, *COX3* gene mutations causing COX3 functional deficiency and hereditary mitochondrial diseases such as Leber hereditary optic neuropathy (LHON), encephalopathy, or myopathy have been reported only rarely. Most of these severe mitochondrial diseases with documented COX3 functional deficiency are caused by mutations in mitochondrial tRNA genes [30,31], suggesting that due to heteroplasmy of *COX3* gene variants, mere *COX3* gene variants may not cause severe, lethal mitochondrial diseases. Although mitochondrial *COX3* gene variants are not necessarily lethal, they could affect survival. Mice with *COX3* gene mutation (G9384A) had markedly poorer physical performance, more mitochondrial superoxide production in brain slices as stained by MitoSox, a mitochondria-targeted fluorogenic dye, and shorter survival than the control mice [32]. Likewise, our study found that *COX3* gene NS variants were associated with poorer prognosis of IPF patients.

We also observed that IPF patients had more mitochondrial *tRNA* variants located in the AC arm, AC loop, variable loop, T-arm, and T loop (the right half of the clover leaf secondary structure) than the control group. Human mtDNA contains 22 genes coding for 20 mitochondrial tRNAs [17]. Although tRNA genes account for only 10% of the mitochondrial genome, most hereditary mitochondrial diseases are caused by pathogenic mutations in mitochondrial tRNA genes [33,34]. The vital importance of mitochondrial tRNA is due to that part of the core subunits of complex I, III, IV, and V are translated through the function of mitochondrial tRNAs.

Previously reported pathogenic mitochondrial tRNA mutations are associated with severe hereditary diseases, affecting organs with high energy demand including nervous system, heart, and skeletal muscles. However, phenotypes of mitochondrial tRNA gene alterations are heterogeneous due to heteroplasmy of mtDNA, with simultaneous presence of mutant and wild-type tRNAs. Our patients were mostly elderly. Their mitochondrial *tRNA* variants were not lethal but can be harmful. Human mitochondrial tRNAs are composed of 65 to 73 nucleotides (shorter and more fragile than cytoplasmic tRNAs), which form a clover-leaf-like secondary structure by Watson–Click base pairing in stem regions (Figure 2) [35] and an L-shaped, folded tertiary structure [36]. The stable tertiary structure is vital for the interaction between mitochondrial tRNA and the aminoacylation enzymes which catalyze the attachment of amino acid to tRNA [17].

We found that compared with the control subjects, IPF patients had more tRNA variants located in the right half of the clover-leaf structure, i.e., in AC stem, the AC loop, variable loop, T-arm, and the T loop. Previous studies have shown that mutations in the AC stem of mitochondrial tRNA for isoleucine (tRNA^Ile^), and those in the AC stem of mitochondrial tRNA for leucine (tRNA^Leu(UUR)^), decreased the efficiency of aminoacylation (to 100- or 1000-fold less) [37,38]. Mutations in T stem disrupted Watson–Click base-pairing or introduced new unstable base pairing, caused unstable T stem structure and impaired tRNA aminoacylation [35,39]. The influence of the variable loop (between the AC stem and the T stem) mutations can be similar to those of the AC and the T stems, causing alteration of base-pairing in the AC or the T stem and structural instability. As for the AC loop, any mutation in this anticodon region can directly affect ability of tRNA to read the codon in mRNA. Thus, mutations in these right-half regions of secondary tRNA structure can affect stability, structure, and function of mitochondrial tRNAs, synthesis of the complex I, III, IV, and V enzymes, efficiency of the ETC, and contribute to the risk and prognosis of IPF patients.

Multivariate regression analysis discovered that older age, current smoker, initial FVC < 60% of predicted value, initial HRCT fibrotic score > 8, *COX* NS variants, and *tRNA* mutation were associated with poor survival. The first three factors align with findings from previous studies; however, we did not identify male gender as a poor prognostic factor, contrary to earlier reports [40,41,42]. Furthermore, we were unable to assess if pulmonary hypertension or co-existing lung cancer as potential poor prognostic factors, due to the lack of echocardiography data for all IPF patients and the absence of lung cancer cases in our cohort [40].

To our knowledge, no previous studies have reported an association of mtDNA variants and IPF prognosis. Although elevated mtDNA concentrations in plasma [15] and bronchoalveolar lavage fluid [43] have been linked to increased IPF mortality, it remains unclear whether these elevated mtDNA concentrations are directly related to mtDNA variants. We examined only mtDNA variants in PBLs. Whether pulmonary epithelial cells of IPF patients have these mtDNA variants is elusive. In a mouse experiment, intra-tracheally instilled ultrafine particles have been shown to cause basement membrane gaps and allow particles to enter the capillary and blood circulation [18]. PBLs can then be exposed to these ultrafine particles and affected. Other potential mechanisms that may cause mtDNA variants in PBLs include chronic inflammation, hypoxia, pulmonary hypertension [44], or genetic factors [20]. These factors have been associated with mitochondrial dysfunction and mtDNA changes in peripheral blood monocytes among patients with chronic obstructive pulmonary disease (COPD), bronchial asthma, or pulmonary hypertension, with unclear mechanism [44]. Whether mitochondrial dysfunction or mtDNA changes in peripheral blood monocytes influence pulmonary cells is unknown.

Our study has both originality and limitations. It was the first to discover that mtDNA variants in *COX3* and *tRNA* genes were associated with risk and prognosis of IPF patients. The limitations are: (1) We only examined the leukocyte mitochondrial genome; whether these gene alterations occur in the lungs or other organs were elusive; (2) the consequences of these variants were not examined; (3) sample size was small; and (4) the follow-up period was not long. Further study on a larger sample with longer follow-up period is needed.

## 4. Conclusions

Our study demonstrates that PBL (1) mitochondrial *COX3* NS variants are associated with risk (being more common in the IPF patients than the control group) and prognosis (being more common in the succumbed than the surviving patients) of IPF; and (2) mitochondrial *tRNA* variants located in the AC stem, AC loop, variable loop, T-arm, and T loop of the tRNA clover-leaf structure are associated with the risk, and the presence of *tRNA* variants is associated with poor prognosis of IPF.

## 5. Materials and Methods

This prospective case-control study was conducted in Fu Jen Catholic University Hospital (FJUH) according to the guidelines of the Declaration of Helsinki and approved by the Institution Review Board of the Fu Jen Catholic University Hospital (FJUH109035). All participants gave informed written consent.

### 5.1. Patient and Control Subject Recruitment

From August 2020 to December 2022, patients treated at the FJUH for IPF were proposed to participate in the study. IPF diagnosis was based on consensus guidelines [2] with cough, dyspnea, basal rales, HRCT findings compatible with usual interstitial pneumonitis (UIP) including interstitial fibrosis, honeycomb appearance, traction bronchiectasis in sub-pleural region and/or lower lobes, and restrictive pattern of pulmonary function with decreased DLCO. The extent of interstitial lung disease on HRCT as quantitative fibrosis score was evaluated by a radiologist. Briefly, each lung was divided into upper (apex to aortic arch), middle (aortic arch to inferior pulmonary vein) and lower (inferior pulmonary vein to lung base) lung zone. The extent of abnormalities in each zone was scored 0–4, while 0 = no abnormality, 1 = 1–25%, 2 = 26–50%, 3 = 51–75%, and 4 = 76–100%. The final score was the sum of 6 zones [45].

Control subjects who were between 50 and 95 years old and did not have IPF, active malignancy, neurodegenerative, liver, kidney, heart, psychiatric, or infectious diseases were recruited at FJUH. All the participants were followed until September 2023 or death.

### 5.2. Blood Sampling and Follow-Up

Genomic DNA was extracted from peripheral blood leukocytes (Puregene Blood Core Kit C, QIAGEN, Hilden, Germany). Participants were evaluated every 1–3 months. Lung function and DLCO were followed every 6–12 months, and chest HRCT every one to two years. Treatment for IPF patients was decided by each patient’s attending physician without any intervention from the investigators. The progression of IPF was defined as: (1) a decline of ≥10 percentage points in the predicted FVC, or (2) death [46,47]. Patients without IPF progression were defined as stable.

### 5.3. Sequencing of the Entire Mitochondrial Genome Using Next-Generation Sequencing

The mitochondrial genome was amplified from leukocyte genomic DNA using mitochondrial target primers with a specific tag to produce a 7.7 and a 9.2 kb fragments [48,49]. The PCR products entered a 2-step PCR to generate bar-coded amplicons (Bar-coded Universal Primers, PacBio, Menlo Park, CA, USA) for SMRTbell library (SMRTbell Express template prep kit 2.0, PacBio, Menlo Park, CA, USA). The SMRT sequencing was performed on the PacBio Sequel sequencer. Sequence reads were aligned to the standard revised Cambridge reference sequence (rCRS NC_012920.1, Homo sapiens mitochondrion complete genome) for comparison.

### 5.4. Statistical Analysis

Quantitative data were shown as mean ± standard deviation and compared using *t*-test. Percentages of cases with a certain variant were compared using z-test. Association between mortality and various factors was analyzed using chi-squared test. Survival curves were generated and compared using log-rank test. Risk factors associated with mortality were analyzed by Cox proportional hazards regression. All analyses were performed using SAS (Version 9.2, SAS Institute Inc., Cary, NC, USA).

## Figures and Tables

**Table 1:** Clinical characteristics of patients with IPF and controls.

Variable	IPF (n = 36)	Control (n = 80)	*p*
Age (years)	75.5 ± 8.4	72.3 ± 9.0	0.073
Female	7 (19)	22 (28)	0.453
Race (Taiwanese * or Chinese **), No. (%) of Taiwanese	30 (83)	70 (88)	0.752
Never smoker	15 (42)	45 (56)	0.232
Heavy alcohol use ***	5 (14)	6 (8)	0.505
Co-existing diseases			
Hypertension	16 (44)	48 (60)	0.175
Diabetes mellitus	15 (42)	31 (39)	0.920
Coronary artery disease	9 (33)	16 (20)	0.188
Stroke	2 (6)	11 (14)	0.313
Heart failure	6 (17)	4 (5)	0.078
Malignancy	5 (14)	7 (9)	0.641
Connective tissue disease	0	4 (5) ^†^	0.415
Chronic kidney disease	2 (6)	5 (6)	0.783
Liver cirrhosis	2 (6)	0	0.175
Initial fibrosis score on HRCT	8.6 ± 2.3 [6–14]		
Initial FVC (as % predicted)	75.4 ± 26.2 [32–192]		
Initial DLCO (as % predicted)	69.4 ± 24.0 [22–121]		
Anti-fibrotic treatment			
Nintedanib only	15 (42)		
Perfenidone only	8 (22)		
Two drugs sequentially	11 (31)		
No anti-fibrotic treatment	2 (5)		
Clinical course			
Progression	21 (58)		
Stable	15 (42)		
Mortality	12 (33)	6 (8)	0.001
Cause of death			
IPF progression	11	0	
Septic shock ^#^	1	1	
Pneumonia	0	3	
Stroke	0	2	

Data were number (%), mean ± standard deviation, or [min–max]. Abbreviations: CKD: chronic kidney disease; DLCO: diffusion capacity for carbon monoxide; FVC: forced vital capacity; HRCT: high-resolution computerized tomography. * Non-indigenous Taiwanese ** Chinese Han people who, or whose parents, were born in China and migrated to live in Taiwan. *** Consuming ≥ 210 gm of alcohol per week for men; ≥112 gm of alcohol per week for women. ^†^ Including pemphigus in 2 and one patient each for Sjogren’s syndrome and autoimmune thyroiditis. ^#^ The one in the IPF group had diabetic foot and the one in the control group had pemphigus.

**Table 2:** *COX3* gene non-synonymous variants (substitution/deletion/insertion) in the IPF and the control group.

Position in mtDNA	rs Number	Variant Classification	Allele Change	Amino Acid Change	Zygosity	No. in the IPF Group	No. in the Control Group
9468	rs879015841	SNS	Acc/Gcc	T/A	Homo *	0	1
9477	(-)	Insertion	gtt/gTtt	V/VX	Hetero ^ǂ^	5	2
9490	(-)	SNS	gCa/gTa	A/V	Homo	1	0
9682	rs199750417	SNS	aTa/aCa	M/T	Homo	1	0
9794	(-)	Deletion	Ttt/tt	F/X	Hetero	2	1
9894	(-)	SNS	Aaa/Gaa	K/E	Homo	0	1
9910	(-)	SNS	tTc/tCc	F/S	Hetero	0	1
9957	rs1556423753	SNS	Ttt/Ctt	F/L	Homo	0	1
9966	rs200809063	SNS	Gtc/Atc	V/I	Homo	0	1
Total Number						9	8
Mutation/deletion/insertion						2/2/5	5/1/2
*p* value						0.240	

* Homoplasmy. ^ǂ^ Heteroplasmy; all variants with heteroplamy showed 50% heteroplasmy. Abbreviations: A, alanine; E, glutamic acid; F, Phenylalanine; I, isoleucine; L, leucine; K, lysine; M, methionine; NS, non-synonymous; S, serine; SNS, single nucleotide substitution; T, threonine; V, valine; X: frameshift variant (deletion or insertion) that leads to change of codes for all downstream amino acids.

**Table 3:** Mitochondrial tRNA gene variants in the IPF and control group.

Variant Position in mtDNA	tRNA	rs Number	Variant (Allele Change)	No. in the IPF Group/Location/ Zygosity	No. in the Control Group/Location/ Zygosity
593	F	rs879123694	SNS (T/C)	0	2/D loop/homo
629	F	rs201031012	SNS (T/C)	0	1/T loop/homo
1664	V	rs200807305	SNS (G/A)	1/A stem/hetero	1/A stem/homo
3290	L1	rs199474665	SNS (T/C)	1/T loop/homo	0
4270	I	(-)	SNS (T/C)	0	1/A stem/hetero
4353 (*ls*)	Q	(-)	SNS (T/C)	0	1/T-arm/homo
5601 (*ls*)	A	rs376884056	SNS (C/T)	1/T loop/homo	0
5673 (*ls*)	N	rs386828975	SNS (T/C)	1/T loop/homo	0
5692 (*ls*)	N	rs199476131	SNS (T/C)	1/AC loop/hetero	0
5773 (*ls*)	C	rs9659239	SNS (G/A)	0	2/T-arm/homo
5814 (*ls*)	C	rs200077222	SNS (T/C)	1/D arm/homo	0
5821 (*ls*)	C	rs200587831	SNS (G/A)	1/A stem/homo	2/A stem/homo
7492 (*ls*)	S1	rs879080411	SNS (C/T)	0	1/AC arm/homo
7521	D	rs200336937	SNS (G/A)	0	3/A stem/homo
7527	D	(-)	Insertion (G/GA)	0	1/D arm/hetero
10047	G	(-)	Deletion (CA/C)	2/T-arm/hetero	0
10410	R	rs200478835	SNS (T/C)	0	1/A stem/homo
10427	R	rs1556423809	SNS (G/A)	0	1/AC arm/homo
10448	R	(-)	SNS (T/C)	1/Variable loop/homo	0
10454	R	rs878874133	SNS (T/C)	1/T loop/homo	0
10463	R	rs28358279	SNS (T/C)	0	1/A stem/homo
12152	H	(-)	Insertion (A/AT)	0	1/D loop/hetero
12153	H	rs376606918	SNS (C/T)	1/D loop/homo	0
12192	H	rs3134560	SNS (G/A)	1/T loop/homo	0
12216	S2	(-)	SNS (C/T)	0	1/D arm/homo
12234	S2	(-)	SNS (A/G)	1/AC arm/homo	0
12239	S2	rs376062400	SNS (C/T)	1/Variable loop/homo	0
12279	L2	(-)	SNS (A/G)	0	1/D loop/hetero
12280	L2	(-)	SNS (A/G)	0	1/D loop/hetero
12280	L2	(-)	SNS (A/G)	0	1/D loop/homo
14710 (*ls*)	E	(-)	Insertion (G/GA)	1/AC loop/hetero	0
14727 (*ls*)	E	(-)	SNS (T/C)	1/D loop/homo	0
15889	T	rs199833246	SNS (G/A)	1/A stem/hetero	0
15891	T	rs1556424681	SNS (C/T)	0	2/A stem/homo
15901	T	(-)	SNS (A/G)	0	1/D loop/homo
15914	T	(-)	SNS (A/G)	0	1/AC arm/homo
15927	T	rs193303002	SNS (G/A)	1/AC arm/homo	1/AC arm/homo
15928	T	rs527236198	SNS (G/A)	1/AC arm/homo	0
15940	T	rs879197567	SNS (T/C)	0	1/T loop/hetero
15940	T	rs879197567	SNS (T/C)	1/T loop/homo	0
16000 (*ls*)	P	rs1556424722	SNS (G/A)	1/AC arm/homo	0
Total number				22	29
A stem				3	11
D arm				1	2
D loop				2	7
AC arm				4	4
AC loop				2	0
Variable loop				2	0
T-arm				2	3
T loop				6	2
AC arm + AC loop + Variable loop + T-arm + T loop				16 (73%)	9 (31%), *p* = 0.008
Light strand				8	6
Mutation/deletion/insertion				19/2/1	27/0/2

Abbreviations: A, alanine; C, cysteine; D, aspartic acid; E, glutamic acid; F, phenylalanine; G, glycine; H, histidine; I, isoleucine; L1, leucine (UUR); L2, leucine (CUN); *ls*, light-strand; M, methionine; N, asparagine; P, proline; Q, glutamine; R, arginine; S1, serine (UCN); S2, serine (AGY); SNS, single nucleotide substitution; T, threonine; V, valine; * all variants with heteroplasmy showed 50% heteroplasmy.

**Table 4:** Clinical characteristics and mtDNA variants of the succumbed and alive patients with IPF.

Characteristics	Succumbed Group (N = 12)	Alive Group (N = 24)	*p*
Age (years)	80.7 ± 6.7 [74–95]	72.9 ± 8.1 [51–86]	0.007
Female	3 (25)	4 (17)	0.881
Never smoker	5 (42)	10 (42)	0.720
Drinker	3 (25)	2 (8)	0.394
Co-existing diseases			
Diabetes mellitus	5 (42)	10 (42)	0.720
Hypertension	7 (58)	9 (38)	0.406
Coronary artery disease	4 (33)	8 (33)	0.708
Heart failure	4 (33)	2 (8)	0.155
Stroke	0	2 (8)	0.797
Malignancy	2 (17)	3 (13)	0.864
Chronic kidney disease	2 (17)	0	0.199
Liver cirrhosis	1 (8)	0	0.720
FVC (% predicted)			
At diagnosis	68.2 ± 19.7 [39–115]	79.1 ± 28.5 [40–192]	0.244
6 months after diagnosis	63.2 ± 24.7 [21–84]	72.6 ± 16.8 [41–106]	0.321
DLCO (% predicted)			
At diagnosis	51.3 ± 23.0 [22–90]	76.5 ± 20.8 [30–121]	0.007
6 months after diagnosis	67.8 ± 34.4 [22–118]	64.9 ± 17.4 [25–96]	0.787
Initial fibrosis score on HRCT	9.7 ± 2.6 [6–14]	8 ± 2.0 [6–13]	0.042
No. (%) of patients with *COX3* NS variants *	5 (42)	3 (13)	0.119
No. of NS variants per patient	0.5 ± 0.7 [0–2]	0.1 ± 0.3 [0–1]	0.032
No. (%) of patients with *tRNA* gene variants	8 (67)	7 (29)	0.069
No. of tRNA variants per patient	1.3 ± 1.2 [0–4]	0.3 ± 0.5 [0–1]	0.002
Anti-fibrotic treatment			
Nintedanib, only	4 (33)	11 (46)	0.925
Pirfenidone, only	2 (17)	6 (25)	
Nintedanib→pirfenidone, or vice versa	5 (42)	6 (25)	
Never	1 (8)	1 (4)	

Data are number (%) or mean ± standard deviation [min–max]. Abbreviation: COX3, cytochrome c oxidase subunit 3; DLCO, diffusion capacity of the lung for carbon monoxide; FVC, forced vital capacity; HRCT, high-resolution computed tomography; NS, nonsynonymous; * Mutation includes substitution, deletion, and insertion.

**Table 5:** Prognostic factors for survival in IPF patients determined by Cox proportional hazards regression analysis.

Risk Factor	*p* Value	Hazard Ratio	95% Confidence Interval
Age	0.016	1.232	1.04–1.4
Sex	0.804	1.371	0.113–16.5
Smoking	0.045		
Current smoker	0.013	32.738	2.09–512
Ever smoker	0.067	6.875	0.87–54.3
Initial FVC < 60% predicted	0.010	11.795	1.81–77.1
Initial HRCT fibrosis score	0.017	118	2.34–5980
*COX3* NS mutation/*tRNA* mutation	0.005		
Either one mutated	0.018	16.426	1.61–167
Both mutated	0.001	245.002	9.15–6560

## Data Availability

Data and materials of the study are available from the corresponding author on reasonable request.

## Supplementary Materials

The following supporting information can be downloaded at: https://www.mdpi.com/article/10.3390/ijms26031378/s1.

## Abbreviations

AC: anticodon; COX3, cytochrome c oxidase subunit 3; DLCO, diffusion capacity of the lung for carbon monoxide; ETC, electron transfer chain; FVC, forced vital capacity; HRCT, high-resolution computed tomography; IPF, idiopathic pulmonary fibrosis; mtDNA, mitochondrial DNA; NS, nonsynonymous; OXPHOS, oxidative phosphorylation system; ROS, reactive oxygen species; TGF-β1, transforming growth factor beta1; tRNA^Ile^, mitochondrial tRNA for isoleucine; tRNA^Leu(UUR)^, mitochondrial tRNA for leucine.
