# Supplementary material for: Mitochondrial COX3 and tRNA Gene Variants Associated with Risk and Prognosis of Idiopathic Pulmonary Fibrosis
Source: Int J Mol Sci. 2025 Feb 6;26(3):1378. doi: 10.3390/ijms26031378 (PMC11818280; doi:10.3390/ijms26031378)
Supplement: Supplementary file 1 [file ijms-26-01378-s001.zip › ijms-3396464-supplementary.pdf]

Table S1. Number of variants and non-synonymous (NS) mutation (substitution/deletion/insertion) in each mitochondrial (mt) gene in patients with idiopathic pulmonary fibrosis (IPF) and control subjects.

Mitochondrial gene, sequence number	IPF (N=36)		Control (N=80)		*p*
	Total	Per patient	Total	Per patient	
NCR, 1…577 and 16024…16569	447	12.4 ± 3.0	1028	12.9 ± 3.3	0.457
TRNF (tRNA phenylalanine), 578…648	0		3		0.586
RRNS (12S ribosomal RNA), 649…1602	94	2.6 ± 1.0	208	2.6 ± 0.9	0.953
TRNV (tRNA valine), 1603…1671	1		1		0.852
RRNL (16S ribosomal RNA), 1672…3229	70	1.9 ± 0.9	145	1.8 ± 0.9	0.451
TRNL1 (tRNA leucine 1, UUA/G), 3230…3304	1		0		0.681
ND subunit 1, 307…4262	43	1.2 ± 1.0	95	1.2 ± 1.2	0.979
NS variants	10	0.3 ± 0.5	26	0.3 ± 0.6	0.686
TRNI (tRNA isoleucine), 4263…4331	0		1		0.681
TRNQ (tRNA glutamine) (Ls), 4329…4400	0		1		0.681
TRNM (tRNA methionine), 4402…4469	0		0		
ND subunit 2, 4470…5511	83	2.3 ± 1.1	209	2.6 ± 1.1	0.172
NS variants	25	0.7 ± 0.7	61	0.8 ± 0.8	0.662
TRNW (tRNA tryptophan), 5512…5576	0		0		
Intron, 5577-5586	2		1		0.472
TRNA (tRNA alanine) (Ls), 5587…5655	1		0		0.681
TRNN (tRNA asparagine) (Ls), 5657…5729	2		0		0.175
Intron (Ls), 5730…5752	2		10		0.420
TRNC (tRNA cysteine) (Ls), 5761…5826	2		4		0.743
TRNY (tRNA tyrosine) (Ls), 5826…5891	0		0		
Intron, 5892…5903	2		6		0.989
COX subunit 1, 5904…7445	80	2.2 ± 1.2	207	2.6 ± 1.2	0.131
NS variants	6	0.2 ± 0.4	12	0.2 ± 0.5	0.551
TRNS1 (tRNA serine 1) (Ls), 7445…7516	0		1		0.681
TRND (tRNA aspartic acid), 7518…7585	0		4		0.415
COX subunit 2, 7586…8269	17	0.5 ± 0.7	37	0.5 ± 0.7	0.942
NS variants	4	0.1 ± 0.3	13	0.2 ± 0.4	0.438
Intron, 8270…8294	6		17		0.748
TRNK (tRNA lysine), 8295…8364	0		0		
ATP subunit 8, 8366…8527	11	0.3 ± 0.7	18	0.2 ± 0.5	0.486
NS variants	7	0.2 ± 0.4	7	0.1 ± 0.3	0.438
ATP subunit 6, 8528…9207	85	2.4 ± 1.1	190	2.4 ± 1.0	0.946
NS variants	73	2.0 ± 1.0	165	2.1 ± 0.8	0.849
COX subunit 3, 9207…9990	42	1.2 ± 1.1	87	1.1 ± 0.9	0.670
NS variants	9	0.3 ± 0.5	8	0.1 ± 0.3	0.048
TRNG (tRNA glycine), 9991…10058	2		0		0.175

ND subunit 3, 10059…10404	51	1.4 ± 1.0	120	1.5 ± 1.1	0.691
NS variants	23	0.6 ± 0.5	49	0.6 ± 0.5	0.802
TRNR (tRNA arginine), 10405…10469	2		3		0.959
ND subunit 4L, 10470…10766	12	0.3 ± 0.6	20	0.3 ± 0.5	0.468
NS variants	4	0.1 ± 0.3	9	0.1 ± 0.3	0.983
ND subunit 4, 10760…12137	84	2.3 ± 1.1	197	2.5 ± 1.3	0.609
NS variants	13	0.4 ± 0.6	41	0.5 ± 0.7	0.249
TRNH (tRNA histidine), 12138…12206	2		1		0.476
TRNS2 (tRNA serine 2), 12207…12265	2		1		0.476
TRNL2 (tRNA leucine 2), 12266…12336	0		3		0.586
ND subunit 5, 12337…14148	101	2.8 ± 1.8	245	3.1 ± 1.7	0.461
NS variants	44	1.2 ± 1.2	105	1.3 ± 1.2	0.707
ND subunit 6 (Ls), 14149…14673	29	0.8 ± 0.7	39	0.5 ± 0.7	0.023
NS variants	15	0.3 ± 0.6	16	0.2 ± 0.4	0.258
TRNE (tRNA glutamic acid) (Ls), 14674…14742	2		0		0.175
Intron, 14743…14746	0		0		
CYTB (Cytochrome b), 14747…15887	173	4.8 ± 2.0	363	4.5 ± 1.9	0.486
NS variants	61	2.5 ± 1.0	123	2.3 ± 0.7	0.126
TRNT (tRNA threonine), 15888…15953	4		6		0.777
TRNP (tRNA proline) (Ls), 15955…16023	1		0		0.681
**Total**	1457	39.9 ± 9.0	3245	40.6 ± 7.3	0.659
**NS mutation**	294	8.9 ± 2.4	701	8.8 ± 2.5	0.796
**tRNA gene variants**	22	0.6 ± 0.9	29	0.4 ± 0.6	0.077

*NS: non-synonymous substitution

Abbreviation: ATP, adenosine triphosphate synthase F0; ND, NADH dehydrogenase;

COX, cytochrome c oxidase; Ls, light strand; NCR, non-coding regulatory region

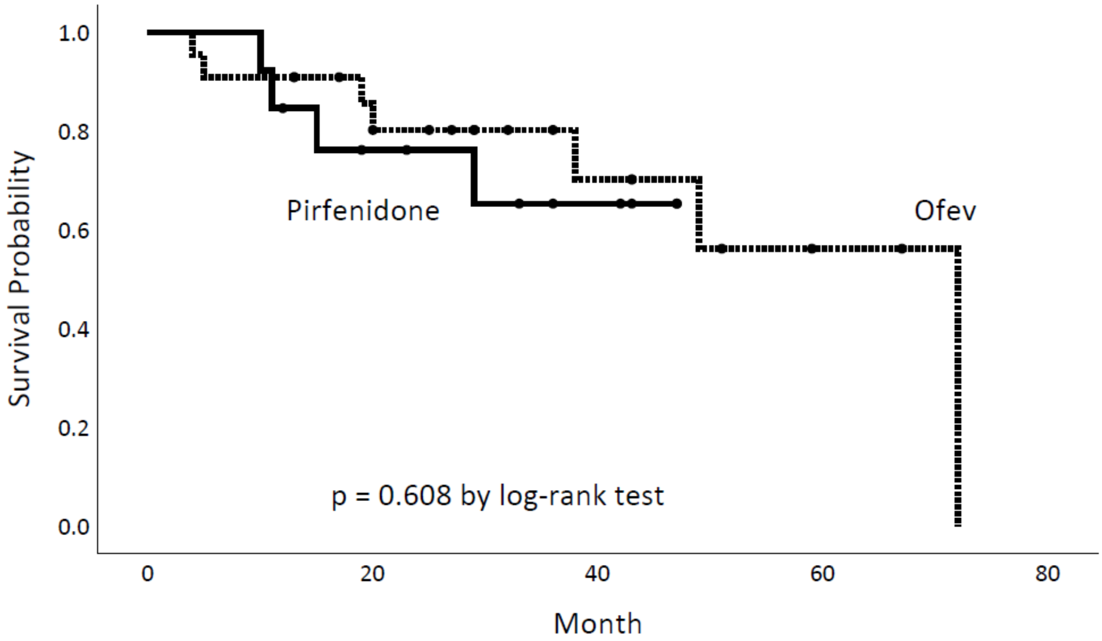

Figure S1

Survival curves of patients with idiopathic pulmonary fibrosis (IPF), who initially received nintedanib (N=23, with or without subsequent change to pirfenidone) and those who initially received pirfenidone (N=13, with or without subsequent change to nintedanib).
